# Supplementary material for: Ganglioside Profiling Uncovers Distinct Patterns in High-Risk Neuroblastoma
Source: Int J Mol Sci. 2025 Aug 29;26(17):8431. doi: 10.3390/ijms26178431 (PMC12428964; doi:10.3390/ijms26178431)
Supplement: Supplementary file 1 [file ijms-26-08431-s001.zip › ijms-3824349-supplementary.pdf]

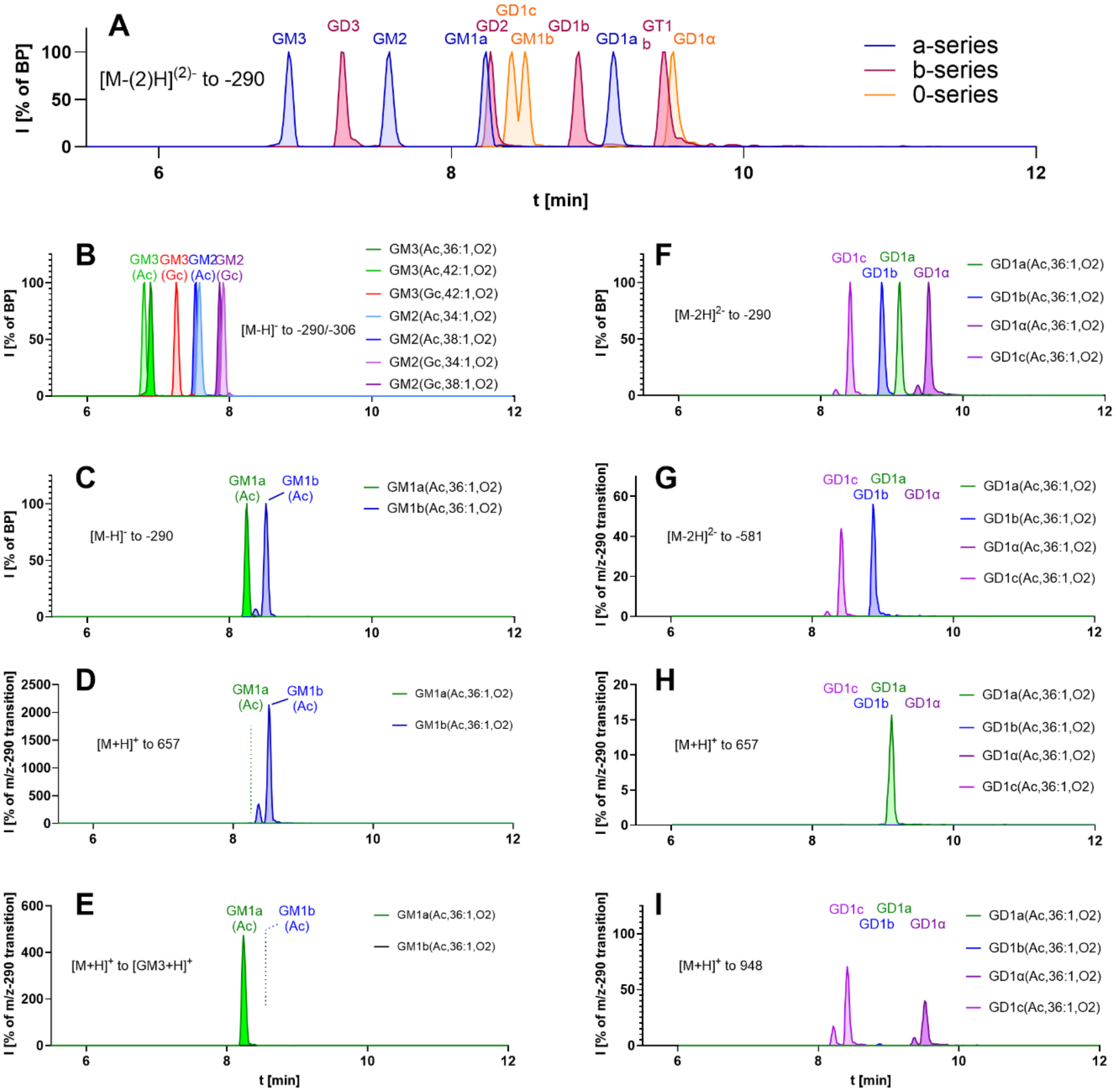

Supplemental Figure S1. HILIC-ESI-MS^2: Chromatographic retention and mass spectrometric transitions of analyzed gangliosides. A, B, C, and F) For quantification all gangliosides were detected with the transition of deprotonated molecular ions ([M-H]- for GM3, GM2, GM1a, GM1b, GD3 and [[M-2H]2- for GD2, GD1a, GD1b, GD1c, GD1alpha, and GT1b) to the sialic acid fragment ion (m/z -290.1 for NeuNAc and m/z 306.1 for NeuNGc). B) Gangliosides with the same glycan but different ceramide anchor chain length elute only slightly different whereas changing N-acetyl to N-glycolyl neuraminic acid results in a rather strong back shift as demonstrated for GM3 and GM2. C) Monosialylated tetraosylceramides LM1, GM1a and GM1b perform identical mass spectrometric transition for quantification but are separated on the HILIC system. D) Qualifier transition to a Hex_HexNAc_NeuNAc fragment in +ESI confirms LM1 and GM1b and excludes GM1a. E) GM1a can be confirmed with the qualifier transition to corresponding [GM3+H]$^+$ fragment in +ESI. F) The identical quantifier transition detects all GD1 species, which are separated by HILIC. G) GD1c and GD1b can be confirmed further with the qualifier transition to a NeuNAc-NeuNAc fragment ion. H) Out of the four GD1 species only GD1a will yield a decent qualifier signal with a transition to a Hex_HexNAc_NeuNAc fragment ion in +ESI. I) Detection of a Hex_HexNac_2NeuNAc ion in +ESI supports the presence of GD1c or GD1α, excluding GD1b and GD1a.

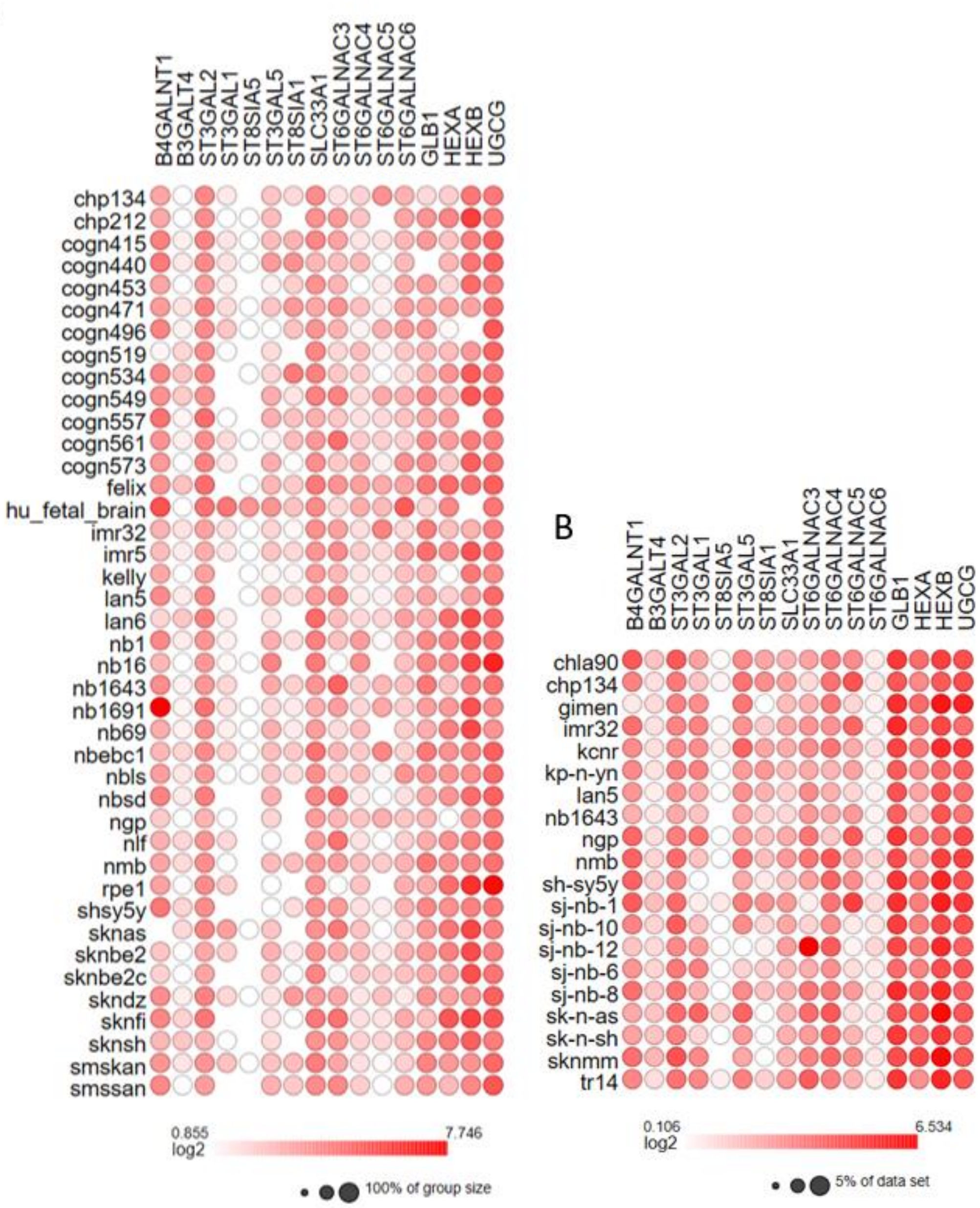

Supplemental Figure S2. Transcriptome profiles of selected GG genes in NBL cell lines. The data indicate a downregulation of *B4GALNT1* in several cell lines, including GI-ME-N and SK-N-AS. A) dataset from GSE89413 (n=41) and B) dataset “Cell line Neuroblastoma – Molenaar” (n=20) via R2 platform.

Supplemental Table S1. Mass spectrometric multi reaction monitoring (MRM) parameters

Quantifier Transitions			
Ganglioside	Precursor	Product ion [m/z]	Collision Energy [eV]
GM3	$[M-H]^-$	-290.1	50
GM3 with NeuNGc	$[M-H]^-$	-306.1	50
GM2	$[M-H]^-$	-290.1	55
GM2 with NeuNGc	$[M-H]^-$	-306.1	55
GM1a, GM1b, LM1	$[M-H]^-$	-290.1	65
GD3	$[M-H]^-$	-290.1	55
GD2	$[M-H]^{2-}$	-290.1	33
GD1a, GD1b, GD1c, GD1α	$[M-H]^{2-}$	-290.1	35
GT1b	$[M-H]^{2-}$	-290.1	32
Qualifier Transitions			
Ganglioside	Precursor	Product ion [m/z]	Collision Energy [eV]
GM1a	$[M+H]^+$	$[GM3+H]^+$	26
GM1b	$[M+H]^+$	+657.2	26
GD1b, GD1c	$[M-H]^{2-}$	-581.2	33
GD1a	$[M+H]2^+$	+657.2	8
GD1c, GD1 α	$[M+H]^+$	+948.3	30
GT1b	$[M-H]^{2-}$	-581.2	35

Supplemental Table S2. Gangliosides concentration as measured by Mass Spectrometry

Patient	GM1b	GD1alpha	GD1c	GM3	GM2	GM1a	GD1a	GD3	GD2	GD1b	GT1b
#1	0,00	0,00	0,00	0,46	0,10	0,11	0,30	0,66	0,70	0,86	3,36
#2	0,00	0,02	0,00	0,26	0,15	0,04	0,46	1,10	0,75	0,63	4,14
#3	0,00	0,00	0,00	0,14	0,14	0,02	0,23	0,62	0,81	0,16	1,79
#4	0,00	0,00	0,00	0,31	0,22	0,05	0,24	1,33	1,11	0,47	2,43
#5	0,00	0,00	0,00	0,97	0,07	0,01	0,04	2,04	1,24	0,20	1,57
#6	0,00	0,01	0,00	0,08	0,10	0,06	0,62	0,23	0,09	0,14	1,97
#7	0,00	0,00	0,00	1,01	0,23	0,07	0,34	1,38	0,92	0,16	1,78
#8	0,00	0,01	0,00	0,51	0,20	0,15	0,20	1,57	2,04	0,85	8,56
#9	0,00	0,01	0,00	0,26	0,17	0,01	0,04	0,62	0,90	0,12	0,65
#10	0,00	0,00	0,00	0,77	0,08	0,00	0,28	0,25	0,05	0,00	0,15
#11	0,00	0,00	0,00	0,29	0,30	0,02	0,12	0,78	1,43	0,17	0,72
#12	0,00	0,00	0,00	0,26	0,22	0,00	0,03	0,62	0,80	0,05	0,10
#13	0,00	0,00	0,00	0,19	0,02	0,06	0,03	0,56	0,12	0,04	0,33
#14	0,00	0,00	0,00	0,50	2,56	1,24	3,34	0,22	1,75	0,39	2,06
#15	0,00	0,01	0,00	0,64	0,21	0,11	0,27	0,54	1,08	0,68	4,19
#16	0,00	0,01	0,00	0,25	0,18	0,28	0,87	0,35	0,18	0,08	1,36
#17	0,00	0,00	0,00	0,06	0,11	0,02	0,06	0,06	0,39	0,07	0,32
#18	0,00	0,00	0,00	0,20	0,74	0,24	1,03	0,10	0,15	0,02	0,23
	0-series			a-series				b-series			

Supplemental Table S3. Correlation between MYCN amplification and GG profiles

	**Profiles**				
**MYCN**	A	B	C	D	E
A	0	2	1	1	0
NA	1	2	1	1	1

Chi-square test: χ^2 = 1.67, p-value = 0.797. A: amplified; NA: not amplified

Supplemental Table S4. Correlation between MYCN amplification and ceramide length

	**Ceramide lenght**			
**MYCN**	long	n.d	normal	short
A	1	0	2	1
NA	3	1	1	1

Chi-square test: χ^2 = 2.01, p-value = 0.570. A: amplified; NA: not amplified

Supplemental Table S5. Cox Proportional Hazard on gene expression

Species	Data type	Category	Tissue/Tumc	Author	N	Normalizatic	Platform	Composition	Material	Accession	Reporter	beta	HR	HR.low	HR.high	wald	pvalue
hs	Expression data	Tumor	Neuroblasto	Westermann	144 total<br>139 with survival data<br>**124 with disease_class: initial**	tpm	genecode19	bulk	RNA	EGAD00001006625	ENSG00000135454.9	-0.4	0.67	0.41	1.1	2.7	0.099
hs	Expression data	Tumor	Neuroblasto	Bell	97 total<br>**95 with survival data**	tmm	ensh38e98	bulk	RNA	GSE181582	ENSG00000135454	-0.32	0.72	0.48	1.1	2.3	0.13
hs	Expression data	Tumor	Neuroblasto	SEQC	498	RPM	seqcnb1	bulk	RNA	GSE62564	NM 001478	-0.42	0.66	0.52	0.84	12	0.00067

Metanalysis result:

```
          HR        95%-CI %W(common) %W(random)
Westermann 0.6700 [0.4100; 1.1000]       15.0       15.0
Bell       0.7200 [0.4800; 1.1000]       21.3       21.3
SEQC       0.6600 [0.5200; 0.8400]       63.7       63.7

Number of studies: k = 3

                         HR           95%-CI     z  p-value
Common effect model  0.6739 [0.5565; 0.8160] -4.04 < 0.0001
Random effects model 0.6739 [0.5565; 0.8160] -4.04 < 0.0001

Quantifying heterogeneity (with 95%-CIs):
 tau^2 = 0 [0.0000; 0.0491]; tau = 0 [0.0000; 0.2215]
 I^2 = 0.0% [0.0%; 89.6%]; H = 1.00 [1.00; 3.10]

Test of heterogeneity:
    Q d.f. p-value
 0.13    2  0.9383

Details of meta-analysis methods:
- Inverse variance method
- DerSimonian-Laird estimator for tau^2
- Jackson method for confidence interval of tau^2 and tau
- Calculation of I^2 based on Q
```

Stratified for HR

Species	Data type	Category	Tissue/Tumc	Author	N	Normalizatic	Platform	Composition	Material	Accession	Reporter	beta	HR	HR.low	HR.high	wald	pvalue
hs	Expression data	Tumor	Neuroblasto	Westermann	144 total<br>139 with survival data<br>**124 with disease_class: initial**<br>**79 with nb2004: hr**	tpm	genecode19	bulk	RNA	EGAD00001006625	ENSG00000135454.9	-0.14	0.87	0.52	1.5	0.29	0.59
hs	Expression data	Tumor	Neuroblasto	SEQC	498 total<br>**176 high_risk: yes**	RPM	seqcnb1	bulk	RNA	GSE62564	NM 001478	-0.19	0.83	0.57	1.2	1	0.31

Metanalysis result:

```
          HR        95%-CI %W(common) %W(random)
Westermann 0.8700 [0.5200; 1.5000]       33.1       33.1
SEQC       0.8300 [0.5700; 1.2000]       66.9       66.9

Number of studies: k = 2

                         HR           95%-CI     z  p-value
Common effect model  0.8430 [0.6217; 1.1431] -1.10  0.2718
Random effects model 0.8430 [0.6217; 1.1431] -1.10  0.2718

Quantifying heterogeneity:
 tau^2 = 0; tau = 0; I^2 = 0.0%; H = 1.00

Test of heterogeneity:
    Q d.f. p-value
 0.02    1  0.8867

Details of meta-analysis methods:
- Inverse variance method
- DerSimonian-Laird estimator for tau^2
- Calculation of I^2 based on Q
```
